# Graphical Abstract: ChemistryOpen 3/2015

**DOI:** 10.1002/open.201580311

**Published:** 2015-06-11

ChemistryOPEN is a multidisciplinary, gold-road, open-access, international forum for the publication of Reviews, Full Papers and Communications from all areas of chemistry and related fields. ChemistryOpen also publishes the Thesis Treasury containing summaries of Ph.D. theses and links to the full version via our homepage. Based in Europe, ChemistryOpen attracts authors and readers from around the world, as open-access publishing becomes more important in all areas of chemistry. ChemistryOpen is coowned by ChemPubSoc Europe and published by Wiley-VCH. Authors can submit their review articles, primary research articles and thesis summaries via our homepage by clicking “submit an article”. All contributions considered suitable for publication are subject to peer review, and if accepted, electronically processed and published online ensuring high quality and short publication times.

## COVER PICTURE

**Figure:**
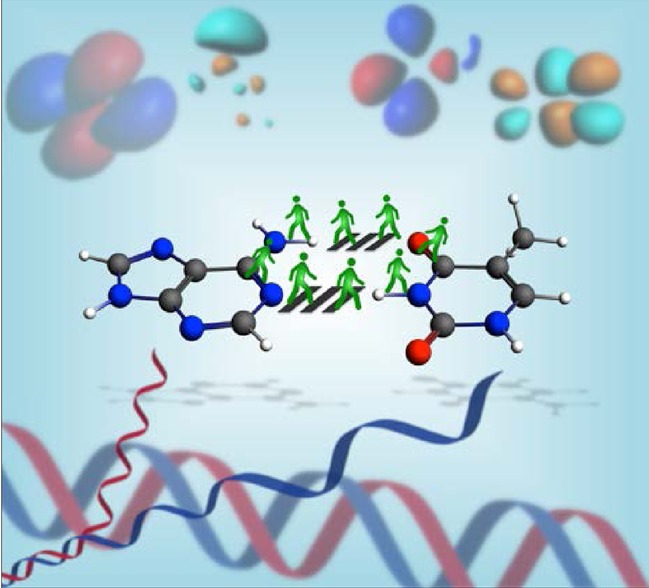


The cover picture shows hydrogen bonds in the adenine–thymine (AT) Watson– Crick base pair. An essential part of these H-bonds is their covalent component that arises from donor–acceptor interactions between the N or O lone pairs and the N–H antibonding s* acceptor orbital. This charge-transfer interaction is represented by the green figures walking over the pedestrian crossing, connecting the two bases. We reveal that this covalent component is the reason why H-bonds between DNA bases and/or unsaturated model bases are significantly stronger than those between analogous saturated bases. This contrasts sharply with the classical picture of predominantly electrostatic H-bonds which is not only incomplete in terms of a proper bonding mechanism, but also fails to explain the trend in stability. For more details, see the Full Paper on p. 318 ff.

## COVER PROFILE

**Figure:**
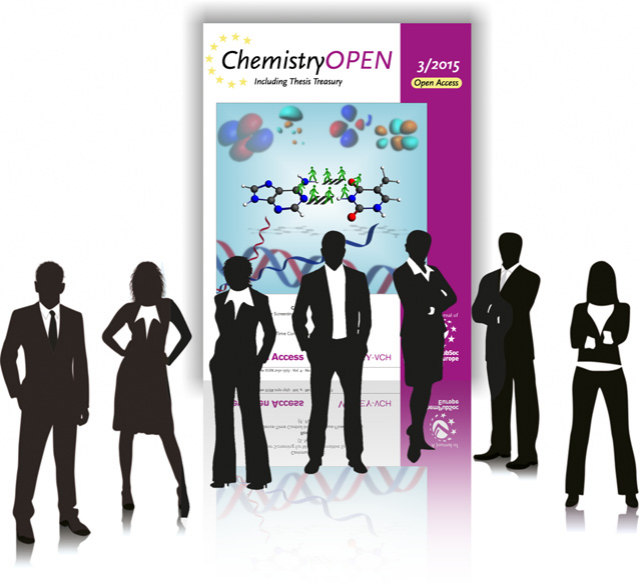


“Our quantum chemical bonding analyses shatter a textbook model. We prove resonance-assisted hydrogen bonding is not the reason for the enhanced H-bond strength between two sp2-hybridized monomers, such as adenine and thymine.” Learn more about the story behind the research featured on the front cover in this issue’s Cover Profile. Read the corresponding article on p. 318 ff.

The Role of Aromaticity, Hybridization, Electrostatics, and Covalency in Resonance-Assisted Hydrogen Bonds of Adenine–Thymine (AT) Base Pairs and Their Mimics

## NEWS

Spotlights on our sister journals 208 – 211

## REVIEWS

**Figure:**
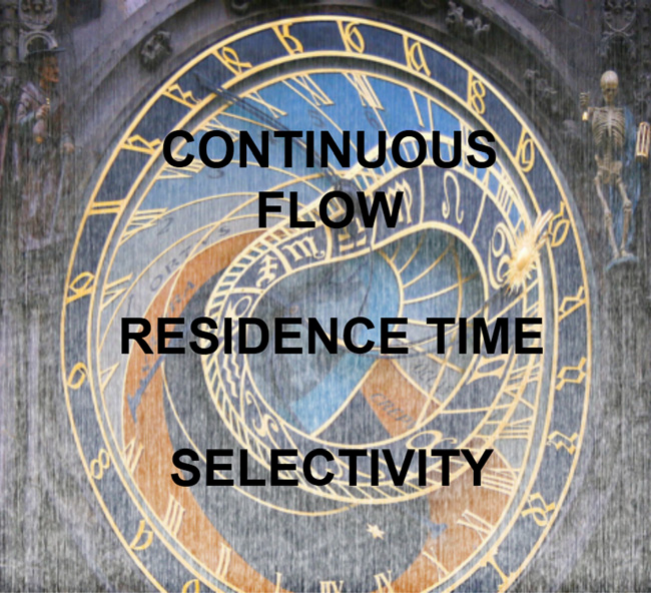


Strategic Application of Residence- Time Control in Continuous-Flow Reactors

**Let it flow!** Residence time is one of the key parameters of continuous-reaction technology, as it directly influences reaction rate, conversion, and product selectivity. This review furnishes a brief insight into continuous-flow reactions in which high chemo- and/or stereoselectivity is attained by strategic residencetime control and illustrates the importance of the residence time as a crucial parameter in sustainable method development.

Recent Advances in Voltammetry

**Figure:**
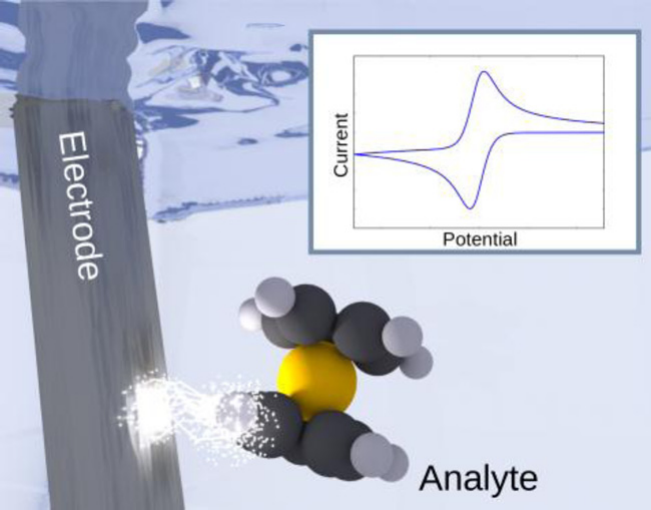


**Visions of voltammetry!** This review covers the major developments made in the last five to seven years in the use and theoretical understanding of voltammetric techniques for studying interfacial processes. Areas of contemporary interest are prioritised with the inclusion of both molecular and nanoparticulate studies.

## COMMUNICATIONS

**Figure:**
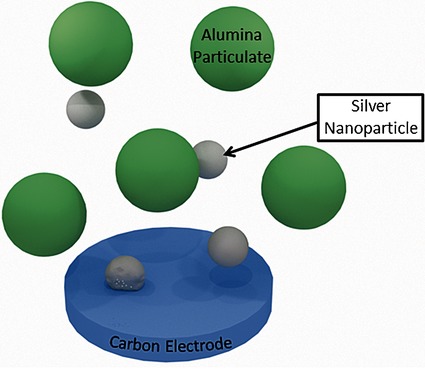


‘Nano-impacts’: An Electrochemical Technique for Nanoparticle Sizing in Optically Opaque Solutions

**Small events with big impact!** The ‘nano-impacts’ technique was shown to be a feasible method for silver nanoparticle detection and size determination in optically opaque medium, such as a solution containing a large amount of alumina, where tradition in situ methods fail. This confers the ‘nano-impacts’ technique a strong advantage over optical methods that are unable to process optically opaque samples.

**Fluorescent flippers:** Mixed oligomers containing dithienothiophene S,S-dioxides, thieno[3,4]pyrazines and 2,1,3- benzothiadiazoles were designed, synthesized and evaluated for their potential use as “fluorescent flippers” in mechanosensitive membrane probes.

**Figure:**
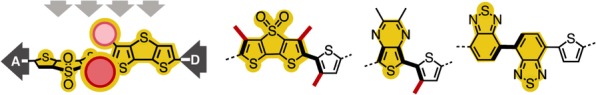


Design and Synthesis of Mixed Oligomers with Thiophenes, Dithienothiophene S,S-Dioxides, Thieno[3,4]pyrazines and 2,1,3- Benzothiadiazoles: Flipper Screening for Mechanosensitive Systems

**A composite material** comprised of monodispersed platinum nanoparticles on high-quality graphene was synthesized using two different exfoliating techniques. The material’s performance as a catalyst in the electrocatalytic production of hydrogen from water at neutral pH is subsequently evaluated. The material exhibited a remarkably high turnover frequency at zero overpotential (∼4600 h¢1 at pH 6.8). The material has potential in the development of robust and scalable water-splitting devices.

**Figure:**
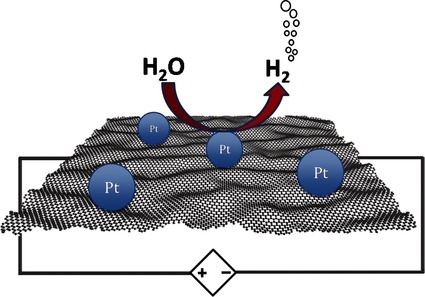


Uniform Functionalization of High-Quality Graphene with Platinum Nanoparticles for Electrocatalytic Water Reduction

**Size and shape matter!** Precise control of the morphology of functional mirco/ nanomaterials could allow for the control of their performance. Fe4(OH)3(PO4)3 microcrystals of different morphologies were successfully prepared under hydrothermal conditions by changing the reaction time, temperature, or amount of hexadecyltrimethylammonium bromide (CTAB). More importantly, their magnetic properties were affected by their size and shape.

**Figure:**
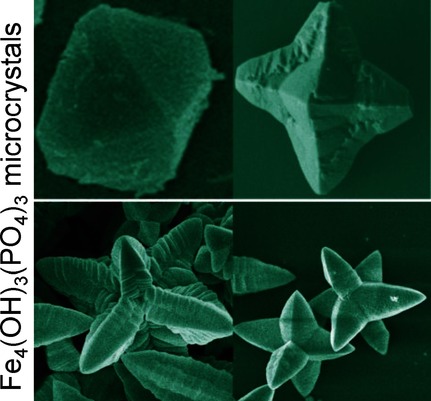


Ferric Phosphate Hydroxide Microstructures Affect Their Magnetic Properties

## FULL PAPERS

**Figure:**
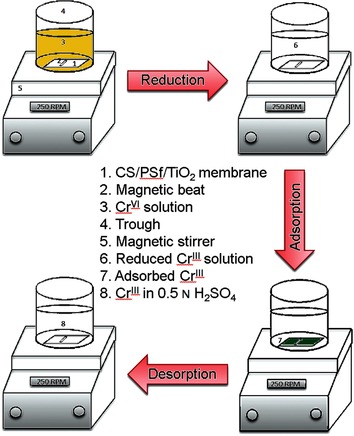


**Cleaning up!** Chitosan thin films were developed on polysulfone/TiO2 composite membranes and were characterized by IR spectroscopy, XRD, SEM, contact angle measurement, and water uptake studies. The resulting membranes were used for the reduction of toxic CrVI to CrIII, which can then be adsorbed to the surface. This technique will facilitate the removal of toxic CrIV species from polluted water sources.

Preparation and Characterization of Chitosan Thin Films on Mixed-Matrix Membranes for Complete Removal of Chromium

Surfactant-Free and Controlled Synthesis of Hexagonal CeVO4 Nanoplates: Photocatalytic Activity and Superhydrophobic Property

**Figure:**
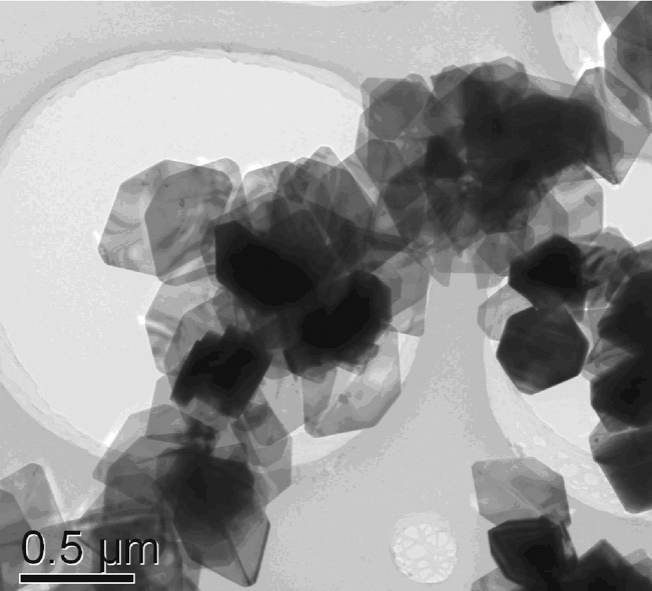


**Nanoplates to go!** Nanomaterials with superhydrophobic surface properties as well as photocatalytic activities could have important applications. CeVO4 hexagonal nanoplates were synthesized under simple and mild conditions. Solutions of the nanoparticles could photocatalytically degrade rhodamine B dye. The nanoplates were also used to coat glass substrates, forming superhydrophobic surfaces, with contact angles reaching 169.5 8.

Quantum Chemical Calculations and Experimental Validation of the Photoclick Reaction for Fluorescent Labeling of the 5’ cap of Eukaryotic mRNAs

**Figure:**
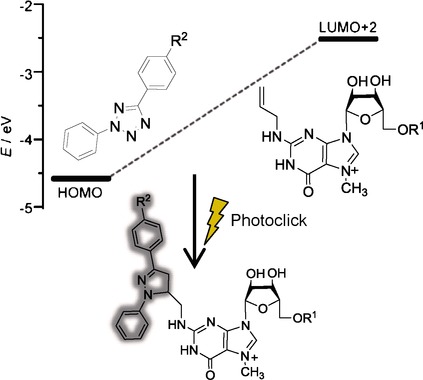


**Clicking with mRNA!** Bioorthogonal click reactions are powerful tools to specifically label biomolecules in living cells. This joint theoretical and experimental study shows that an N-allylmodified 5’ cap found in mRNA can be reacted with tetrazoles in a photoclick reaction. The combination of enzymatic allylation and photoclick chemistry generates a turn-on fluorophore specifically at the 5’ cap found in eukaryotic mRNAs.

Revisiting Aromaticity and Chemical Bonding of Fluorinated Benzene Derivatives

**Figure:**
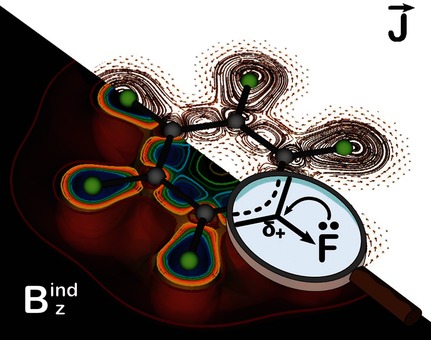


**Determining aromaticity:** Here, we analyzed the electron delocalization in fluorinated benzene derivatives (C6H(6¢n)Fn) in terms of the induced magnetic field, nucleus-independent chemical shift (NICS), and ring current strength (RCS). Fluorination was found to decrease the paratropic ring current through inductive effects and to decrease the diatropic ring current through resonance effects, and the balance between these two effects decreases aromaticity with increased fluorination.

Influence of Polarity and Activation Energy in Microwave–Assisted Organic Synthesis (MAOS)

**Figure:**
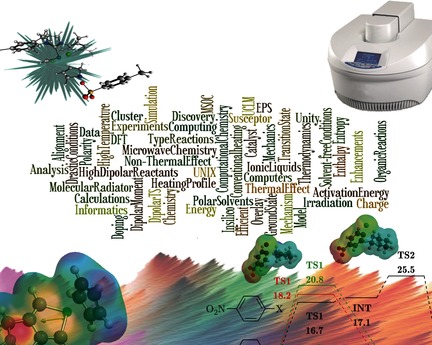


**Mastering microwaves:** This work sought to determine parameters that have decisive roles in microwave-assisted organic reactions (MAOS) and to develop a model, using computational chemistry, to predict the type of reactions that can be improved by using microwave irradiation. Parameters such as polarity, activation energy, and enthalpy were found to be important in the improvement of MAOS.

**σ beats π!** Hydrogen bonds play a crucial role in many biochemical processes. We show quantum chemically that neither aromaticity nor π assistance is responsible for the enhanced stability of the H-bonds in adenine–thymine DNA base pairs. Our bonding analyses reveal that stronger lone pair to σ * N–H donor–acceptor interactions are behind the enhanced and contracted H-bonds between aromatic and other unsaturated model bases, as compared with fully saturated analogs.

**Figure:**
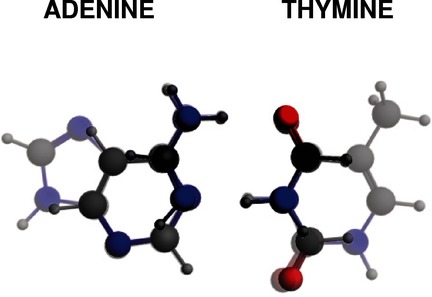


The Role of Aromaticity, Hybridization, Electrostatics, and Covalency in Resonance-Assisted Hydrogen Bonds of Adenine–Thymine (AT) Base Pairs and Their Mimics

**Nickel-based catalysis:** Nickel(II) complexes make up a new generation of olefin catalysts which are important for processes like ethylene polymerization. Novel 4-arylimino-1,2,3-trihydroacridylnickel( II) dihalide complexes were synthesized in a one-pot reaction and characterized. Upon activation with trimethylaluminium (TMA), all complexes exhibited good activity for ethylene oligomerization and products ranged from butene (C4) to hexadecene (C16).

**Figure:**
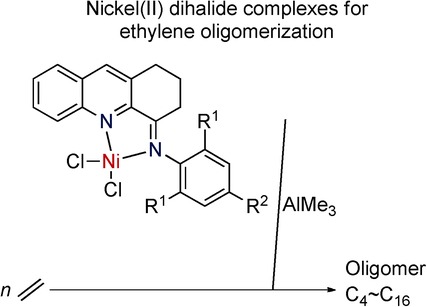


Nickel(II) Complexes Bearing 4- Arylimino-1,2,3-trihydroacridines: Synthesis, Characterization, and Ethylene Oligomerization

**Targeting tumors via epigenetics:** Histone deacetylase inhibitors (HDACi) alter the epigenetic state of tumors and are promising therapeutics for cancer. Although studies with HDACi have shown promise in some cancers, variable efficacy and off-target effects have limited their use. Here we report the design and evaluation of a tumor-specific dendrimer– HDACi.

**Figure:**
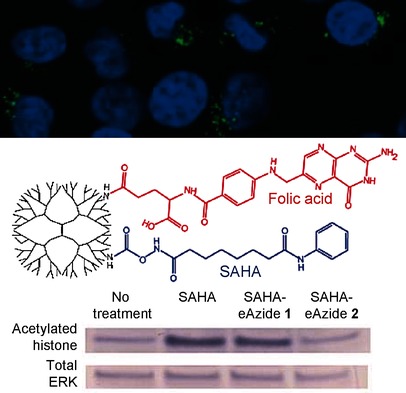


Design and Evaluation of Tumor-Specific Dendrimer Epigenetic Therapeutics

**Targeting tuberculosis:** 5-styryl-oxathiazol- 2-ones were found to be inhibitors of the Mycobacterium tuberculosis (Mtb) proteasome. The compounds displayed a good selectivity for Mtb and were rapidly bactericidal against nonreplicating Mtb. The results suggest that this new class of Mtb proteasome inhibitors has the potential to be further developed into novel antitubercular agents for synergistic combination therapies with existing drugs.

**Figure:**
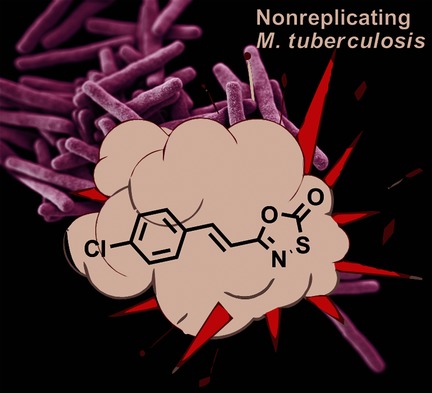


Optimization and Evaluation of 5-Styryl-Oxathiazol-2-one Mycobacterium tuberculosis Proteasome Inhibitors as Potential Antitubercular Agents

Colorimetric Cyanide Chemosensor Based on 1’,3,3’,4-Tetrahydrospiro[ chromene-2,2’-indole]

**Figure:**
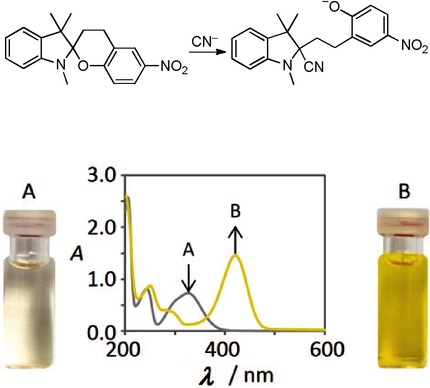


**Cyanide before your eyes!** A new type of cyanide chemosensor based on the 1’,3,3’,4-tetrahydrospiro-[chromene-2,2’- indole] scaffold is reported. These compounds show a distinct color change visible to the naked eye when treated with cyanide and exhibit high sensitivity, selectivity, and fast response within tens of seconds.

Trichocyanines: a Red-Hair-Inspired Modular Platform for Dye-Based One- Time-Pad Molecular Cryptography

**Figure:**
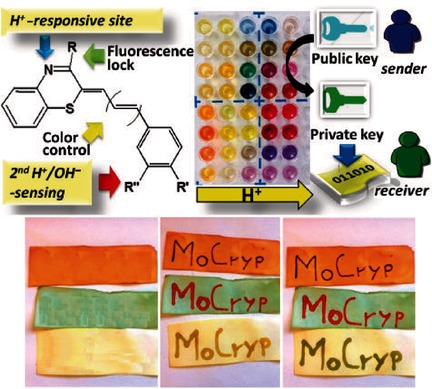


**Color-coded chemical cryptography:** A versatile dye platform could generate an expandable palette of colors specifically suited to implement an unprecedented single-use asymmetric molecular cryptography (MoCryp) system. Eight representative acidichromic cyaninetype dyes were used in the system. The trichocyanine dyes, originally inspired by red hair pigments, were pH-sensitive and tunable through four different control points.

A New Fluorescence Turn-On Probe for Aluminum(III) with High Selectivity and Sensitivity, and its Application to Bioimaging

**Figure:**
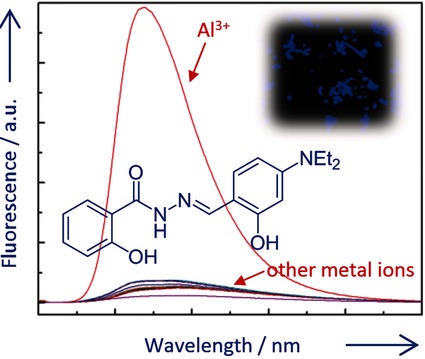


**Sensing Al!** Selective, sensitive fluorescent probes for Al3+ are need given the importance of this metal in biological processes. Herein, a readily available turn-on fluorescent chemosensor is reported, which displays highly selective and sensitive Al3+-amplified fluorescence emission over other common metal ions and is able to detect Al3+ in E. coli via bioimaging.

## THESIS TREASURY

Supported Sulfonic Acids: Solid Catalysts for Batch and Continuous-Flow Synthetic Processes

**Figure:**
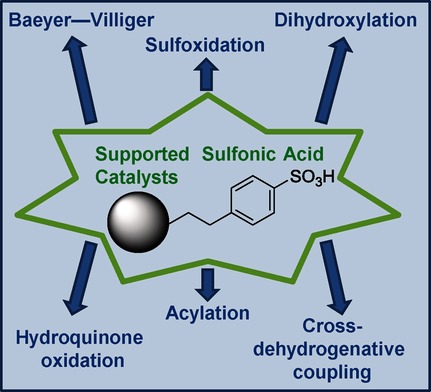


**It’s easy being green!** In this work, supported sulfonic acids were used as effective catalysts for different classes of reactions. They have the advantage of being cheap, safe, and easy to recycle. Reaction conditions are mild, have good atom economy, and work-up steps are very easy, satisfying essential parameters of the “green chemistry” philosophy. Application of these catalysts to continuous-flow processes resulted in a considerable improvement in efficiency.

**Antimicrobial peptides**(AMPs) were investigated both as novel antibiotics and as antimicrobial coatings for biomedical implants. The hydrophobicity, conformational constraint, and strong binding of cyclo-RRRWFW (c-WFW) to the O-antigen region of lipopolysaccharide (LPS) were identified as important features for potent anti-E. coli activity. Furthermore, tethered membrane-active AMPs with uniform distribution of cationic and hydrophobic amino acid residue were identified as good anti-biofilm agents.

**Figure:**
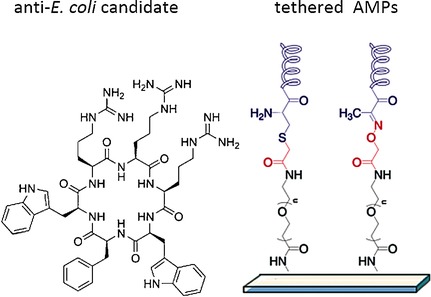


Cationic Antimicrobial Peptides (AMPs): Thermodynamic Characterization of Peptide–Lipid Interactions and Biological Efficacy of Surface-Tethered Peptides

## SERVICE

*Author to whom correspondence should be addressed.

Supporting information is available on the WWW

This is an open-access article, published under the terms and conditions of a Creative Commons License, as stated in the final article. (see article for access details).

A video clip is available as Supporting Information on the WWW (see article for access details).

Contributions labeled with this symbol have been judged as “Very Important Papers” by the referees.

